# Comparison of IHC, FISH and RT-PCR Methods for Detection of *ALK* Rearrangements in 312 Non-Small Cell Lung Cancer Patients in Taiwan

**DOI:** 10.1371/journal.pone.0070839

**Published:** 2013-08-07

**Authors:** Yi-Cheng Wu, Il-Chi Chang, Chi-Liang Wang, Tai-Di Chen, Ya-Ting Chen, Hui-Ping Liu, Yen Chu, Yu-Ting Chiu, Tzu-Hua Wu, Li-Hui Chou, Yi-Rong Chen, Shiu-Feng Huang

**Affiliations:** 1 Department of CardioVascular and Thoracic Surgery, Chang Gung Memorial Hospital at Linko, and Chang Gung University College of Medicine, Taoyuan, Taiwan; 2 Institute of Molecular and Genomic Medicine, National Health Research Institutes, Miaoli, Taiwan; 3 Division of Pulmonology, Department of Medicine, Chang Gung Memorial Hospital at Linko, Chang Gung University College of Medicine, Taoyuan, Taiwan; 4 Department of Pathology, Chang Gung Memorial Hospital at Linko, Taoyuan, Taiwan; 5 Institute of Cancer Research, National Health Research Institutes, Miaoli, Taiwan; 6 Department of Pathology, Buddhist Tzu-Chi Medical Center, Taipei Branch, New Taipei, Taiwan; 7 Department of Medicine, Tzu-Chi University College of Medicine, Hualien, Taiwan; Health Canada and University of Ottawa, Canada

## Abstract

**Background:**

Recently Echinoderm microtubule-associated protein-like 4- anaplastic lymphoma kinase (*EML4-ALK*) fusion gene has become an important biomarker for ALK tyrosine kinase inhibitor (crizotinib) treatment in NSCLC. However, the best detection method and the significance of *EML4-ALK* variant types remain uncertain.

**Methods:**

Reverse transcriptase-polymerase chain reaction (RT-PCR), fluorescence in Situ hybridization (FISH) and Immunohistochemical (IHC) stain were performed on tumor tissues of 312 NSCLC patients for detection of *ALK* rearrangements. Mutation analyses for *EGFR* and *KRAS* genes were also performed.

**Results:**

Thirteen of the 312 patients (4.17%) had *ALK* rearrangements detected by RT-PCR. If RT-PCR data was used as the gold standard, FISH tests had a low sensitivity (58.33%), but very good specificity (99.32%). IHC stain had better sensitivity (91.67%) than FISH, but lower specificity (79.52%), when the cut off was IHC2+. All of the 8 patients with high abundance of *EML4-ALK* positive cells in tumor tissues (assessed by the signal intensities of the RT-PCR product), were also have high expression of ALK protein (IHC3+), and positive for FISH, except one failed in FISH. Variants 3a+3b (4/5, 80%) of *EML4-ALK* fusion gene were more common to have high abundance of *EML4-ALK* positive cells in tumor tissues than variant 1 (1/3, 33.3%). Meta-analysis of the published data of 2273 NSCLC patients revealed that variant 3 (23/44, 52.3%) was the most common type in Chinese population, while variant 1 (28/37, 75.7%) was most common in Caucasian.

**Conclusions:**

Among the three detection methods, RT-PCR could detect not only the presence of *EML4-ALK* fusion gene and their variant types, but also the abundance of *EML4-ALK* positive cells in NSCLC tumor tissues. The latter two factors might affect the treatment response to anti-ALK inhibitor. Including RT-PCR as a diagnostic test for ALK inhibitor treatment in the prospective clinical trials is recommended.

## Introduction

Lung cancers, especially non-small cell lung cancers (NSCLC) has been the leading cause of cancer death in the world, and the patient number is still increasing [1,2]. The great success of targeted therapy with epidermal growth factor receptor (EGFR)-tyrosine kinase inhibitor (TKI): gefitinib and erlotinib, for treatment of lung adenocarcinoma has made targeted therapy become the most popular modality for major human cancers [3–7]. Searching for new and effective targets other than EGFR in lung cancer treatment has not been successful until year 2007, when Soda, et al identified the echinoderm microtubule- associated protein-like-4 and the anaplastic lymphoma kinase (*EML4-ALK*) fusion gene with transforming ability in NSCLC patients [8]. ALK protein is a 200kDa receptor tyrosine kinase. Its signal pathway involves cell proliferation, differentiation, and anti-apoptosis [8,9]. Before the identification of *EML4-ALK* fusion gene, *Nucleophosmin*-*ALK* (NPM-ALK) fusion gene was first identified in anaplastic large cell lymphoma [10]. Various combinations of ALK with other proteins has been discovered in different neoplasms, such as diffuse large B cell lymphoma, inflammatory myofibroblastic tumor, neuroblastoma, squamous cell carcinoma of the esophagus, and renal cell carcinoma [11–13]. But the *EML4-ALK* fusion gene is unique to NSCLC [14]. ALK is now recognized as an important oncogenic driver in NSCLC. Multiple *EML4-ALK* variants in NSCLCs have been identified, all contain the same C-terminal kinase domain, and confer gain-of-function properties [14]. Although *EML4* gene is the predominant fusion partner of *ALK* in NSCLC, other fusion partner genes also have been identified, which included *KIF5B-ALK, KLC1-ALK and TFG-ALK* fusion genes [15–18]. Majority of *ALK* rearrangements were identified in adenocarcinoma and non-smokers [8,14,18–28]. The incidences of *EML4-ALK* fusion gene in NSCLC were around 1.4 to 11.6% with no significant differences between Asian and western countries [8,14,18–28]. While *EML4-ALK* fusion was identified in NSCLC, a new ALK tyrosine kinase inhibitor (ALK-TKI): crizotinib was developed at similar time. This drug originally was designed as a MET tyrosine kinase inhibitor for treatment of NSCLC patients, but it was found to be an ALK inhibitor, too. Thus, prospective clinical trials were designed and started quickly on NSCLC patients with *EML4-ALK* fusion genes. The phase II clinical trial was published in year 2010, which demonstrated dramatic response and longer progression free survival to crizotinib treatment in patients with *EML4-ALK* fusion gene [29–31], similar to EGFR–TKI on NSCLC patients with *EGFR* mutations [3–7]. Crizotinib was approved by the Food and Drug Administration (FDA) of the United States in year 2011 for treatment of NSCLC patients, and with a companion diagnostic test, the Vysis ALK Break Apart FISH Probe Kit, that could help determine if a patient has the abnormal *ALK* gene [32]. *EML4-ALK* fusion gene becomes a new important molecular marker for crizotinib treatment in NSCLC patients. However, the best detection method for *EML4-ALK* fusion gene remains controversial. Theoretically, reverse transcriptase-polymerase chain reaction (RT-PCR) and fluorescence in situ hybridization (FISH) are two standard methods for detection of fusion genes, but both have significant limitations in clinical practice. RT-PCR requires fresh frozen tissue samples for extraction of RNA, and a reliable FISH assay requires good fluorescence scope and technical expertise. Immunohistochemical (IHC) stain for detection of ALK over-expression has been a well-established method for detection of *ALK* rearrangements, such as NPM-ALK, in hematological malignancies for years [33,34]. Since the cost of IHC stain assay is much lower than that of FISH assay, IHC stains could be a much more convenient and cost-effective screening method for *ALK* rearrangements in NSCLCs, when compared with FISH or RT-PCR. However, the sensitivity of IHC stain remained controversial. Many research groups reported good correlations between IHC stain and FISH for detection of *EML4-ALK* fusion gene [16,35–38], but some reported that IHC stain was non-sensitive in detection of *EML4-ALK* fusion gene [25,39–41]. It would be most ideal to perform all three methods (IHC, FISH and RT-PCR) in a large series of NSCLC tumors for direct comparison of their efficacies.

Previously, we have reported a good correlation between IHC stain for ALK and the RT-PCR study for identification of *EML4-ALK* fusion genes in 64 NSCLC patients [42]. We have extended our study to 312 NSCLC patients and performed all three methods (IHC, FISH, and RT-PCR) for direct comparison of the sensitivity and specificity.

## Results

### *ALK* rearrangement detected by RT-PCR

The clinic-pathological characteristics of the 312 patients are shown in Table S1. Among the 312 patients included for the RT-PCR study, thirteen patients (13/312, 4.17%), three male (one was never smoker) and ten female (seven were never smokers), were found to have *ALK* rearrangement. Twelve had *EML4-ALK* fusion gene and one had *KIF5B-ALK* fusion gene. The pathology diagnoses were adenocarcinomas (ADC) in eleven patients, squamous cell carcinoma (SCC) in one patient, and adenosquamous carcinoma (ADSC) in one patient. None of the 13 patients had co-existing *EGFR* or *KRAS* mutations. The incidence of *ALK* rearrangement in EGFR-wild type, non-SCC patients was 12% (12/100). *KLC1-ALK*, and *TFG-ALK* fusion genes were not identified. Among the 12 patients with *EML4-ALK* fusion gene, three were variant 1, one was variant 2, five were variant 3a+3b with 3b predominant, two were variant 3a, and one was variant 3b. *ALK* rearrangement was only significantly associated with female gender (P= 0.0248) by univariate analysis. Although majority (8/13, 61.5%) of the patients with *ALK* rearrangement were in stage I and none was in stage IV, the association with tumor stage was statistically non-significant (p= 0.5740). Multivariate analysis for the association with five clinico-pathological characteristics, i.e. gender, age, smoking history, histology types and tumor stage, was performed by binary logistic regression test. In addition to female gender (p=0.007), non-smoker also became significantly associated with *ALK* rearrangements (p=0.022), since there was a big difference in the smoking rate between male and female patients (never smokers were 38% in male versus 94.4% in female, p<0.0001). The tumor stage remained non-significant. The P values for each stage were: Stage I: P= 0.6220, Stage II: P= 0.4040, Stage III: P= 0.6190, and Stage IV: P= 0.9990, respectively.

### *ALK* rearrangement determined by FISH

Among the 312 patients, FISH studies were not performed on two patients, since no residual tumor tissue could be found in the paraffin sections. FISH studies failed in another 5 patients. As a result, FISH analysis data was available in a total of 305 patients. Nine patients (9/305, 2.95%) were positive by break apart FISH study. All of them had adenocarcinoma. One was male patient (current smoker), and eight were female patients (two were current smokers). Only 7 of the 9 patients were also RT-PCR (+).

### ALK protein expression detected by IHC stain

A total of 310 patients had successful IHC stain for detection of ALK protein expression, since 2 patients without tumor part in the tissue sections were excluded. All of the 78 patients with negative IHC stain were RT-PCR (-) and FISH (-). For the 159 patients with IHC 1+, only one patient was RT-PCR (+), but FISH (-), It was the only one SCC positive for RT-PCR. For the 61 patients with IHC 2+, three patient was RT-PCR (+), but FISH (-), and one patients was RT-PCR (-) but FISH (+). For the 12 patients with IHC 3+, nine were RT-PCR (+). Seven of them were also FISH (+), which included the only one patient with *KIF5B-ALK* fusion gene (Figure S1). Another one was FISH (-) and the last one failed in FISH. For the remaining 3 patients with IHC3+ and RT-PCR (-), one was FISH (+) (Figure S2), one was FISH (-), and one failed in FISH. The latter 2 patients also had EGFR mutations.

### EGFR mutation analyses

Among the 312 NSCLC patients, the *EGFR* mutation rate was 43.91% (137/312). All were non-SCC, which included 133 ADC and 4 ADSC. The *EGFR* mutation rate in non-SCC patients was 57.81% (137/237). For non-SCC patients, *EGFR* mutations were only significantly associated with never smokers (p=0.0002), but not with age or gender. None of the patients had co-existing *ALK* or *KRAS* mutations.

### KRAS mutation analyses

Among the 312 NSCLC patients, the *KRAS* mutation rate was 5.77% (18/312), which included 16 adenocarcinomas and two adenosquamous carcinomas. The mutation rate in non-SCC was 7.59% (18/237). Twelve patients were male (10 were smokers) and 6 were female (none were smokers). *KRAS* mutations were only significantly associated with the histology type (non-squamous cell carcinoma) (p=0.0091).

### Comparison of the three detection methods

A total of 305 patients had data of all three detection methods (RT-PCR, FISH and IHC) for direct comparison. The results are shown in Table S2. The study results of all 17 patients positive for either RT-PCR, or FISH or high ALK expression were summarized in Table S3. The histological features of all 17 patients were reviewed. All adenocarcinomas positive for ALK were composed of mixed histological patterns.

### The specificity and sensitivity of FISH and IHC according to the RT-PCR data

If RT-PCR results were used as the gold standard for ALK rearrangement, and the cut-off for ALK protein expression was IHC 2+, the sensitivity and specificity of IHC was 91.67% and 79.52%, respectively. The positive predictive value of IHC was only 15.49% and the negative prediction value was 99.57%. If the cut-off was IHC 3+, the sensitivity and specificity of IHC was 66.67% and 99.32%, respectively. The positive predictive value of IHC was 80.00% and the negative prediction value was 98.64%. For the FISH test, though its sensitivity was only 58.33%, the specificity was very high (99.32%). The positive predictive value of FISH was 77.78% and the negative prediction value was 98.31%.

### Assessment of the abundance of EML4-ALK positive cells in tumor tissues by RT-PCR

The abundance of *EML4-ALK* positive cells in tumor tissues was assessed according to the intensities of the RT-PCR products on gel electrophoresis. The result of each tumor was compared with the FISH and IHC data (Table S3). Eight of the 13 RT-PCR(+) tumors had strong intensity of the RT-PCR products, which suggested high abundance *of EML4-ALK* positive cells in the tumor tissues. All of these 8 patients were IHC 3+, and FISH (+), except one failed in FISH study. For the 5 patients with weak intensity of the RT-PCR products, which suggested low abundance of *EML4-ALK* positive cells in tumor tissues, all of them were FISH (-) and only one was IHC 3+. The latter was a tumor belonged to Variant 3a+3b. Representative cases were shown in Figure S3. When compared with the variant type data, high abundance of *EML4-ALK* positive cells in tumor tissues were more common in Variant 3a+3b (4/5, 80%) than Variant 1 (1/3, 33.3%) (Table S3).

### Meta-analysis of the ethnicity and the variant types associated with *EML4-ALK* fusion gene

Meta-analysis of eighteen studies (including the present study) that had performed RT-PCR study for detection of *EML4*-*ALK* fusion gene were performed and shown in Table S4 and Table S5 [8,15,16,18–28,40,41,43]. Among the 2273 NSCLC patients with available ethnicity and variant type data for *EML4*-*ALK* fusion gene, Variant 3 (23/44, 52.3%) was the most common type in Chinese population, while Variant 1 (28/37, 75.7%) was most common in Caucasian. The difference was statistically significant for both Variant 1(p= 0.0000) and Variant 3 (p=0.0020). With regard to Japanese patients, Variant 1(11/33, 33%) was also the most common variants, but it was not as dominant as in Caucasian.

## Discussion

In this study, the incidence of *ALK* rearrangements in NSCLC patients in Taiwan was quite low (4.17%) according to the RT-PCR results. For *EGFR*-wild type, non-SCC patients, it was 12% (12/100). *ALK* rearrangement was only significantly associated with female gender and non-smokers by multivariate analyses. The above results were quite similar to previously published reports from Asian or western countries. We found that the stage distribution in NSCLC patients with *ALK* rearrangements were all very similar, i.e. largest number in stage I and none or very few in stage IV, between our study and 4 published reports [21,24,25,27], The reason why it was not significantly associated with the tumor stage was that patients without *ALK* rearrangements also had highest patient number in stage I and lowest in stage IV in all of these 5 studies (only 2 to 14 patients in stage IV). This uneven stage distribution was probably due to the availability of fresh frozen tissues for RT-PCR, which were more available in operable patients (stage I to III), and difficult for stage IV patient. Interestingly the incidences of *ALK* rearrangements in stage III patients were all higher than the other stages in our study and 3 of the above 4 reports [21,24,27]. It would require a large case number to determine whether the incidence of *ALK* rearrangements would increase with the tumor stage, since the incidence of *ALK* rearrangements was quite low.

One important goal of this study was to compare the sensitivity and specificity among the three detection methods (IHC, FISH, and RT-PCR). It revealed that FISH had a low sensitivity (58.33%), but with very good specificity (99.32%). In contrast, IHC stain appeared to have better sensitivity (91.67%) than FISH, but the specificity (79.52%) was much lower, when the cut off was IHC2+. To understand whether the antibodies used would affect the IHC results or not, we have performed IHC stains on all RT-PCR (+) tumors with additional ALK antibodies from different companies, which included 5A4 (Histofine, Nichirei), 5A4(Abcam), 5A4 (Novocastra), and D5F3 (Cell Signaling), to compare their sensitivity and specificity. All of the above ALK antibodies have been used in published reports [15,25,35,36,38–40]. It appeared that when the tumors had high ALK expression (IHC 3+) by the anti-ALK antibody we used (ZAL4), they would also be positive with high intensity by all other anti-ALK antibodies (data not shown). In contrast, if the tumors had only moderate ALK expression (IHC 2+) by ZAL4, these tumors would be totally negative for ALK by the other anti-ALK antibodies. As shown in Table S3, four RT-PCR (+) or FISH (+) tumors in this study were IHC 2+. Thus, ZAL4 appeared to have higher sensitive for detection of ALK expression than other anti-ALK antibodies, but with lower specificity.

We have examined all of the gel electrophoresis pictures of RT-PCR (+) tumors to check if it could be correlated with ALK protein expression level in IHC stain. To our surprise, the RT-PCR results had good correlation, not only with ALK protein expression, but also the FISH data. All of the 8 tumors with strong intensity of the PCR products (suggesting high abundance of *EML4-ALK* positive cells in tumor tissues) also had high expression of ALK protein by IHC stains and all positive for FISH tests, except one failed in FISH (Table S3). This correlation has never been reported before. Since we only evaluated 100 tumor cells in FISH test for each tumor, it was reasonable that high abundance of *EML4-ALK* positive cells in tumor tissues would have higher chance to be positive for FISH. Since FISH has been approved as a reliable diagnostic test for crizotinib (ALK inhibitor) treatment [32], our findings might suggest that high abundance of *EML4-ALK* positive cells in tumor tissues could also be related to the treatment response to ALK inhibitors.

It was also interesting to find that high abundance of *EML4-ALK* positive cells in tumor tissues were more common in Variant 3a+3b (4/5, 80%) than Variant 1 (1/3, 33.3%). High ALK protein expression (IHC3+) was also more common in Variant 3a+3b (5/5, 100%) than Variant 1 (1/3, 33,33%). This was similar to the report by Wallander, et al. They found that there was 100% concordance for the detection of *EML4-ALK* variant 3a+3b by all 3 detection methods (RT-PCR, FISH and IHC), but not for variant 1 in a study with 46 Caucasian patients [41]. Since the most common variant of Caucasian NSCLC patients was Variant 1, it might be one reason why IHC methods were found to be non-sensitive in the reports from some of the western study reports [25,40,41], but had good correlation with FISH in the study reports from Asian countries [35,36,38]. Recently *EML4-ALK* variants were also found to have different sensitivity to ALK inhibitor by in vitro study. Variant 1 was the most sensitive to ALK inhibitor, followed by Variant 2 and Variant 3b (equally sensitive), and the last one was Variant 3a [44]. It would be interesting to check whether the variant types of *EML4-ALK* and the patients’ ethnicity could affect the therapeutic response to ALK inhibitor or not.

Another finding worthy for discussion was the two patients with EGFR mutations and high expression of ALK protein (IHC3+), but negative in RT-PCR study. One of them was FISH (-) and another one failed in FISH. Since co-existence of *ALK* and *EGFR* mutations were very rare, another possible explanation for this association is that the high expression of ALK might be induced by *EGFR* mutations through the interaction of these two kinase proteins. Concurrent activation of ALK and EGFR signaling pathways has been reported in the lung cancer cell lines, and these cell lines could only be suppressed by dual inhibition of both ALK and EGFR [21,45].

In summary, among the three detection methods for ALK rearrangements, RT-PCR could detect not only the presence of *EML4-ALK* fusion gene and the variant types, but also the abundance of *EML4-ALK* positive cells in NSCLC tumor tissues. The latter two factors might affect the treatment response to anti-ALK inhibitor. Including RT-PCR as a diagnostic test for ALK inhibitor treatment in the prospective clinical trials is recommended.

## Material and Methods

### Patients and tissues

Fresh frozen tumor specimens of 328 NSCLC patients (274 patients from May 2002 to April 2006 and 54 patients from October 2009 to May 2012) receiving surgical resection at Chang Gung Memorial Hospital and with signed informed consent were obtained from the tissue bank of Chang Gung Memorial Hospital for RNA extraction and molecular biomarker studies. None of the patients have received ALK inhibitor treatment before. Frozen sections were performed on each fresh frozen lung cancer tissues for determination of tumor % by a pathologist (SFH) prior to the study. Only patients with a tumor % higher than 70% were included for this study. Some tumor tissues with low tumor % and abundant fibrous stroma were microdissected manually under microscope to achieve higher tumor %. All of the patients included also need to have available paraffin sections of the lung tumor for ALK IHC stain and FISH study. Finally, a total of 312 patients had sufficient specimens to get into this study. The clinical data and smoking history were obtained from the medical records. None of the patients have received crizotinib treatment before. This study protocol had been reviewed and approved by the Institutional Review Board of Chang Gung Memorial Hospital and National health Research Institutes. Among the 312 patients, the ALK rearrangement and IHC study results of 64 patients have been published previously [42].

### RNA extraction and complementary DNA synthesis

Fresh frozen tumor tissues were used as starting materials for total RNA extraction by Trizol method (Invitrogen, Carlsbad, Calif.). The RNA quality was verified by gel electrophoresis. Two µg of total RNA was used as started material for complementary DNA (cDNA) synthesis by employed with SuperScript III Reverse Transcriptase (200U/µl, Invitrogen, Carlsbad, Calif.). After reverse transcription reaction was finished, a total volume of 40 µl cDNA was obtained. One µl resulting cDNA was used for further PCR reactions.

### Polymerase Chain Reaction (RT-PCR) for Detection of different ALK rearrangement with different partners

#### *EML4-ALK* fusion gene

RT-PCR combined with Sanger sequencing of PCR products were performed. Two primer sets (shown in Table S6) were designed according to the published reports to identify *EML4-ALK* Variant 1 and Variant Non-1, respectively [8,26,46]. The latter could detect *EML4-ALK* Variant 2 to 7. The thermal cycle conditions were different for Variant 1 and Variant Non-1. For *EML4-ALK* Variant 1, the thermal cycle conditions was preincubation for 4 minutes at 95° C for the initial activation, followed by 35 cycles of denaturation for 30 seconds at 95° C, primer annealing for 30 seconds at 55° C, and elongation for 2 minutes 30 seconds at 72° C. For *EML4-ALK* Variant Non-1, the thermal cycle was: 15 minutes at 95° C, followed by 35 cycles of denaturation for 30 seconds at 95° C, primer annealing for 30 seconds at 66° C, and elongation for 1 minute at 72° C. Estimated PCR product size (base pair) of each variant was listed as following: variant 1 (247 bp), variant 2 (2303 bp), variant 3a (728 bp), variant 3b (761 bp), variant 4 (1664 bp), variant 5a (269 bp), variant 5b (386 bp), variant 6 (1619 bp) and variant 7 (1688 bp).

#### *KIF5B-ALK*, *KLC1-ALK* and *TFG-ALK* fusion genes

The possible existence of *KIF5B-ALK*, *KLC1-ALK* and *TFG-ALK*, were also examined in all patients’ tumors that were negative for *EML4-ALK* fusion gene. The primer sets (shown in Table S6) and PCR condition were designed according to the previously published reports [15–18]. The thermal cycle conditions for *KIF5B-ALK* was 95° C for 4 minutes, followed by 40 cycles of 30 seconds at 95° C, 30 seconds at 50° C, and 2 minutes 30 seconds at 72° C for 40 cycles. For *KLC1-ALK*, it was 95° C for 4 minutes, followed by 40 cycles of 30 seconds at 95° C, 30 seconds at 55° C and 3 minutes at 72° C. For *TFG-ALK*, it was 4 minutes at 94° C, followed by 40 cycles of 45 seconds at 94° C, 45 seconds at 65° C, and 1 minute 30 seconds at 72° C.

After the PCR reaction was finished, total PCR product (20 µl) of each case was used for DNA gel electrophoresis. If any PCR product with expected size was obtained, nucleotide sequencing (Applied Biosystems PRISMR 3730 DNA Analyzer Sequencer) was performed. The nucleotide sequences obtained were verified by BLAST program at NCBI. All positive results were repeated by a second technician for confirmation.

### *ALK* rearrangement determined by break apart FISH study

The FISH detection was done on unstained 5-µm-thick paraffin-embedded formalin-fixed tissue sections. According to the hematoxylin and eosin stain of the same tissue block, the tumor portion on each slide was selected and circled by one pathologist (SFH) prior to the FISH study. The *ALK* probe used was the Vysis ALK Break Apart FISH Probe (Abbott Molecular Inc. Des Plaines, IL, USA). The deparaffinized tissue sections were first pretreated with 100 mM Tris, 50 mM EDTA, pH 7.0 solution in 92° C for 15 minutes, followed by phosphate-buffered saline (PBS) wash, then digested with 300 µl of Digest-all (Zymed, Inc., South San Francisco, CA) at 37° C for 90 to 120 minutes depending on the size of the tissue sections. The digestion was stopped by 10% neutral formalin at room temperature for 1 minute and washed with PBS again. Ten µl of *ALK* probe was applied to each dehydrated and air-dried slide, and denatured at 94° C for 4 minutes, then hybridized overnight at 37° C in VYSIS HYBrite (Abbott Molecular Inc. Des Plaines, IL, USA). Post-hybridization wash was performed with 2x standard saline citrate at 72° C for 5 minutes and then rinsed in PBS with 0.25% Tween 20 (Sigma-Aldrich Co., St. Louis, MO). The slides were then mounted with 10 µl of VECTASHIELD mounting medium (Vector Laboratories, Inc., Burlingame, CA) with 0.1µg/ml of 4',6-diamidino-2- phenylindole. The FISH study results were evaluated with Leica DMR fluorescence micro- scope (Leica Microsystems Nussloch GmbH, Nussloch, Germany). The FISH signals are first evaluated by two experienced technicians independently (THW, YTC) and rechecked by one pathologist (SFH) for those FISH positive tumors. At least 100 non-overlapping and intact tumor nuclei were evaluated. The tumor cells were considered to be positive for FISH study according to the manufacturer’s guidelines, i.e. when the red and green signals were wide apart for more than 2 nuclei or had loss of the paired green signal (individual red signal, IRS). Only tumors with more than 15% of tumor cells having positive break-apart FISH signals were considered to have *ALK* rearrangements. The FISH images were taken by SPOT2 image system (DIAGNOSTIC instruments, Inc., IL).

### IHC stain for ALK protein expression

IHC stain was performed on unstained 4-µm-thick paraffin-embedded formalin-fixed tissue sections. The slides were treated by xyline first for deparaffinization, followed by 95° C antigen retrieval in commercial Tris-base buffer with boric acid and ethylene-diamine- tetraacetic acid (Cell Conditioning 1, Roche, Rotkreuz, Switzerland) for 30 minutes. Then the slides were incubated with primary antibody for ALK (clone ZAL4, Invitrogen, Life Tech Corp, Carlsbad, Calif.) overnight at room temperature with a dilution of 1:500 [16,24]. The detection was performed by the Ventana Discovery XT staining system with ultraView Universal DAB detection kit (Ventana Medical Systems, Inc, Tucson, AZ) according to the manufacturer’s protocol. The IHC stains were evaluated for the expression of ALK in tumor tissue by two pathologists (TDC and SFH) without knowledge of the FISH and RT-PCR results. The expression of ALK in tumor tissue was graded as weak (1+), moderate (2+) and high (3+) according to the intensity as in our previous report [42]. If there was uneven distribution of the intensity, it would be graded according to the highest intensity identified in more than 10% of the tumor tissue.

### Mutation analysis for EGFR and KRAS genes by Sanger sequencing

In order to compare with other molecular biomarkers, *EGFR* and *KRAS* gene mutation studies were also performed on all of the 312 NSCLC patients. The DNA extracted from the fresh frozen tumor tissue was used for mutation analyses. The methods were similar to our previous reports [47,48]. The *EGFR* reference sequence NM005228.3 and the *KRAS* reference sequence NM004985.3 were all from the NCBI database

### Statistic analyses

The differences in the major clinical and molecular features correlated with *ALK* gene arrangement are compared by conventional chi-square association test or Fisher’s exact test (when there is at least a cell frequency less than 5). Binary logistic regression test was performed for multivariate analysis. A two-sided *p* value less than 0.05 was considered statistically significant.

## Supporting Information

Figure S1 The study results of the patient with KIF5B-ALK fusion gene. (**A**) Gel electrophoresis revealed correct size of the RT-PCR product. (**B**) The electrospherogram of KIF5B-ALK fusion gene by direct sequencing. (**C**) The green and red signals were wide apart for more than 2 nuclei by break-apart FISH study. (**D**) The IHC stain for ALK was strong (anti-ALK antibody, 200X).
(TIFF)

Figure S2 The study results of patient No. 148. Her lung tumor was RT-PCR (-), but FISH (+) and IHC 3+ (A) The green and red signals were wide apart for more than 2 nuclei by break-apart FISH study. (B) The IHC stain for ALK was strong (anti-ALK antibody, 200X).
(TIFF)

Figure S3 The intensities of the RT-PCR products of *EML4-ALK* fusion gene detected by gel electrophoresis were compared with the FISH and IHC stain data. **Upper left**: All of the 4 patients (No. 10, 30, 81 and 111) had strong intensities of the RT-PCR products. They were all FISH (+) and IHC 3+. **Upper right**: Two (No. 159 and 179) of the four patients had weak intensities of the RT-PCR products, and the other two patients (No. 173 and 177) were RT-PCR (-). One of them (No. 173) was FISH (+) and IHC 2+. **Lower**: IHC stains for ALK in four patients are shown. (**A**) Patient No. 20 was totally negative for ALK, (**B**) Patient No. 179 (squamous cell carcinoma) was IHC 1+, (**C**) Patient No. 173 was IHC 2+, (**D**) Patient No. 111 was IHC 3+ (anti-ALK antibody, 200X).
(TIF)

Table S1 Clinical characteristics of *ALK* rearrangements detected by RT-PCR in 312 NSCLC patients. (DOCX)

Table S2 Comparison between RT-PCR, FISH and IHC detection methods for *ALK* rearrangement in 305 non-small cell carcinoma patients. (DOCX)

Table S3 Clinicopathological characteristics of 17 non-small cell lung cancer patients with ALK rearrangements or having high ALK expression. (DOC)

Table S4 Meta-analysis of 18 ALK rearrangement study reports in non-small cell lung cancer patients with RT-PCR study data. (DOC)

Table S5 The ethnicities and variant types of EML4-ALK fusion gene among 2273 NSCLC patients. (DOC)

Table S6 Primer sets for detection of ALK rearrangements. (DOCX)

## References

[1] Jemal A, Bray F, Center MM, Ferlay J, Ward E et al. (2011) Global cancer statistics. CA Cancer J Clin 61: 69-90. doi:10.3322/caac.20107. PubMed: 21296855. 21296855 10.3322/caac.20107

[2] Jemal A, Siegel R, Ward E, Hao Y, Xu J et al. (2008) Cancer statistics, 2008. CA Cancer J Clin 58: 71-96. doi:10.3322/CA.2007.0010. PubMed: 18287387. 18287387 10.3322/CA.2007.0010

[3] Mitsudomi T, Morita S, Yatabe Y, Negoro S, Okamoto I et al. (2010) Gefitinib versus cisplatin plus docetaxel in patients with non-small-cell lung cancer harbouring mutations of the epidermal growth factor receptor (WJTOG3405): an open label, randomised phase 3 trial. Lancet Oncol 11: 121-128. doi:10.1016/S1470-2045(09)70364-X. PubMed: 20022809. 20022809 10.1016/S1470-2045(09)70364-X

[4] Mok TS, Wu YL, Thongprasert S, Yang CH, Chu DT et al. (2009) Gefitinib or carboplatin-paclitaxel in pulmonary adenocarcinoma. N Engl J Med 361: 947-957. doi:10.1056/NEJMoa0810699. PubMed: 19692680. 19692680 10.1056/NEJMoa0810699

[5] Maemondo M, Inoue A, Kobayashi K, Sugawara S, Oizumi S et al. (2010) Gefitinib or chemotherapy for non-small-cell lung cancer with mutated EGFR. N Engl J Med 362: 2380-2388. doi:10.1056/NEJMoa0909530. PubMed: 20573926. 20573926 10.1056/NEJMoa0909530

[6] Ellis PM, Blais N, Soulieres D, Ionescu DN, Kashyap M et al. (2011) A systematic review and Canadian consensus recommendations on the use of biomarkers in the treatment of non-small cell lung cancer. J Thorac Oncol 6: 1379-1391. doi:10.1097/JTO.0b013e318220cb8e. PubMed: 21709590. 21709590 10.1097/JTO.0b013e318220cb8e

[7] Lynch TJ, Bell DW, Sordella R, Gurubhagavatula S, Okimoto RA et al. (2004) Activating mutations in the epidermal growth factor receptor underlying responsiveness of non-small-cell lung cancer to gefitinib. N Engl J Med 350: 2129-2139. doi:10.1056/NEJMoa040938. PubMed: 15118073. 15118073 10.1056/NEJMoa040938

[8] Soda M, Choi YL, Enomoto M, Takada S, Yamashita Y et al. (2007) Identification of the transforming EML4-ALK fusion gene in non-small-cell lung cancer. Nature 448: 561-566. doi:10.1038/nature05945. PubMed: 17625570. 17625570 10.1038/nature05945

[9] Chiarle R, Voena C, Ambrogio C, Piva R, Inghirami G (2008) The anaplastic lymphoma kinase in the pathogenesis of cancer. Nat Rev Cancer 8: 11-23. doi:10.1038/nrc2291. PubMed: 18097461. 18097461 10.1038/nrc2291

[10] Palmer RH, Vernersson E, Grabbe C, Hallberg B (2009) Anaplastic lymphoma kinase: signalling in development and disease. Biochem J 420: 345-361. doi:10.1042/BJ20090387. PubMed: 19459784. 19459784 10.1042/BJ20090387 PMC2708929

[11] Bai RY, Dieter P, Peschel C, Morris SW, Duyster J (1998) Nucleophosmin-anaplastic lymphoma kinase of large-cell anaplastic lymphoma is a constitutively active tyrosine kinase that utilizes phospholipase C-gamma to mediate its mitogenicity. Mol Cell Biol 18: 6951-6961. PubMed: 9819383. 9819383 10.1128/mcb.18.12.6951 PMC109278

[12] Chiarle R, Gong JZ, Guasparri I, Pesci A, Cai J et al. (2003) NPM-ALK transgenic mice spontaneously develop T-cell lymphomas and plasma cell tumors. Blood 101: 1919-1927. doi:10.1182/blood-2002-05-1343. PubMed: 12424201. 12424201 10.1182/blood-2002-05-1343

[13] Barreca A, Lasorsa E, Riera L, Machiorlatti R, Piva R et al. (2011) Anaplastic lymphoma kinase in human cancer. J Mol Endocrinol 47: R11-R23. doi:10.1530/JME-11-0004. PubMed: 21502284. 21502284 10.1530/JME-11-0004

[14] Choi YL, Takeuchi K, Soda M, Inamura K, Togashi Y et al. (2008) Identification of novel isoforms of the EML4-ALK transforming gene in non-small cell lung cancer. Cancer Res 68: 4971-4976. doi:10.1158/0008-5472.CAN-07-6158. PubMed: 18593892. 18593892 10.1158/0008-5472.CAN-07-6158

[15] Takeuchi K, Choi YL, Togashi Y, Soda M, Hatano S et al. (2009) KIF5B-ALK, a novel fusion oncokinase identified by an immunohistochemistry-based diagnostic system for ALK-positive lung cancer. Clin Cancer Res 15: 3143-3149. doi:10.1158/1078-0432.CCR-08-3248. PubMed: 19383809. 19383809 10.1158/1078-0432.CCR-08-3248

[16] Wong DW, Leung EL, Wong SK, Tin VP, Sihoe AD et al. (2011) A novel KIF5B-ALK variant in nonsmall cell lung cancer. Cancer 117: 2709-2718. doi:10.1002/cncr.25843. PubMed: 21656749. 21656749 10.1002/cncr.25843

[17] Togashi Y, Soda M, Sakata S, Sugawara E, Hatano S et al. (2012) KLC1-ALK: a novel fusion in lung cancer identified using a formalin-fixed paraffin-embedded tissue only. PLOS ONE 7: e31323. doi:10.1371/journal.pone.0031323. PubMed: 22347464. 22347464 10.1371/journal.pone.0031323 PMC3275577

[18] Shinmura K, Kageyama S, Tao H, Bunai T, Suzuki M et al. (2008) *EML4-ALK* fusion transcripts, but no NPM-, TPM3-, CLTC-, ATIC-, or TFG-ALK fusion transcripts, in non-small cell lung carcinomas. Lung Cancer 61: 163-169. doi:10.1016/j.lungcan.2007.12.013. PubMed: 18242762. 18242762 10.1016/j.lungcan.2007.12.013

[19] Inamura K, Takeuchi K, Togashi Y, Nomura K, Ninomiya H et al. (2008) EML4-ALK fusion is linked to histological characteristics in a subset of lung cancers. J Thorac Oncol 3: 13–17. doi:10.1097/JTO.0b013e31815e8b60. PubMed: 18166835. 18166835 10.1097/JTO.0b013e31815e8b60

[20] Takeuchi K, Choi YL, Soda M, Inamura K, Togashi Y et al. (2008) Multiplex reverse transcription−PCR screening for EML4-ALK fusion transcripts. Clin Cancer Res 14: 6618–6624. doi:10.1158/1078-0432.CCR-08-1018. PubMed: 18927303. 18927303 10.1158/1078-0432.CCR-08-1018

[21] Koivunen JP, Mermel C, Zejnullahu K, Murphy C, Lifshits E et al. (2008) EML4-ALK fusion gene and efficacy of an ALK kinase inhibitor in lung cancer. Clin Cancer Res 14: 4275-4283. doi:10.1158/1078-0432.CCR-08-0168. PubMed: 18594010. 18594010 10.1158/1078-0432.CCR-08-0168 PMC3025451

[22] Inamura K, Takeuchi K, Togashi Y, Hatano S, Ninomiya H et al. (2009) EML4-ALK lung cancers are characterized by rare other mutations, a TTF-1 cell lineage, an acinar histology, and young onset. Mod Pathol 22: 508-515. doi:10.1038/modpathol.2009.2. PubMed: 19234440. 19234440 10.1038/modpathol.2009.2

[23] Lin E, Li L, Guan Y, Soriano R, Rivers CS et al. (2009) Exon array profiling detects EML4-ALK fusion in breast, colorectal, and non-small cell lung cancers. Mol Cancer Res 7: 1466–1476. doi:10.1158/1541-7786.MCR-08-0522. PubMed: 19737969. 19737969 10.1158/1541-7786.MCR-08-0522

[24] Wong DW, Leung EL, So KK, Tam IY, Sihoe AD et al. (2009) The *EML4-ALK* fusion gene is involved in various histologic types of lung cancers from nonsmokers with wild-type EGFR and *KRAS* . Cancer 115: 1723-1733. doi:10.1002/cncr.24181. PubMed: 19170230. 19170230 10.1002/cncr.24181

[25] Martelli MP, Sozzi G, Hernandez L, Pettirossi V, Navarro A et al. (2009) EML4-ALK rearrangement in non-small cell lung cancer and non-tumor lung tissues. Am J Pathol 174: 661-670. doi:10.2353/ajpath.2009.080755. PubMed: 19147828. 19147828 10.2353/ajpath.2009.080755 PMC2630573

[26] Takahashi T, Sonobe M, Kobayashi M, Yoshizawa A, Menju T et al. (2010) Clinicopathologic features of non-small-cell lung cancer with EML4-ALK fusion gene. Ann Surg Oncol 17: 889–897. doi:10.1245/s10434-009-0808-7. PubMed: 20183914. 20183914 10.1245/s10434-009-0808-7

[27] Zhang X, Zhang S, Yang X, Yang J, Zhou Q et al. (2010) Fusion of EML4 and ALK is associated with development of lung adenocarcinomas lacking EGFR and KRAS mutations and is correlated with ALK expression. Mol Cancer 9: 188. doi:10.1186/1476-4598-9-188. PubMed: 20624322. 20624322 10.1186/1476-4598-9-188 PMC2908583

[28] Sanders HR, Li HR, Bruey JM, Scheerle JA, Meloni-Ehrig AM et al. (2011) Exon scanning by reverse transcriptase-polymerase chain reaction for detection of known and novel EML4-ALK fusion variants in non-small cell lung cancer. Cancer Genet 204: 45–52. doi:10.1016/j.cancergencyto.2010.08.024. PubMed: 21356191. 21356191 10.1016/j.cancergencyto.2010.08.024

[29] Kwak EL, Bang YJ, Camidge DR, Shaw AT, Solomon B et al. (2010) Anaplastic lymphoma kinase inhibition in non-small-cell lung cancer. N Engl J Med 363: 1693-1703. doi:10.1056/NEJMoa1006448. PubMed: 20979469. 20979469 10.1056/NEJMoa1006448 PMC3014291

[30] Camidge DR, Bang Y, Kwak EL, Shaw AT, Lafrate AJ et al. (2011) Progression-free survival (PFS) from a phase I study of crizotinib (PF-02341066) in patients with ALK-positive non-small cell lung cancer (NSCLC). J Clin Oncol 29 suppl: abstr 2501

[31] Crinò L, Kim D, Riely GJ, Janne PA, Blackhall FH et al. (2011) Initial phase II results with crizotinib in advanced ALK-positive non-small cell lung cancer (NSCLC): PROFILE. J Clin Oncol 1005: 29 suppl: abstr 7514

[32] Ou SH (2011) Crizotinib: a novel and first-in-class multitargeted tyrosine kinase inhibitor for the treatment of anaplastic lymphoma kinase rearranged non-small cell lung cancer and beyond. Drugs Devel Ther 5: 471-485. PubMed: 22162641. 10.2147/DDDT.S19045 PMC3232174 22162641

[33] Lamant L, Meggetto F, al Saati T, Brugières L, de Paillerets BB et al. (1996) High incidence of the t(2;5)(p23;q35) translocation in anaplastic large cell lymphoma and its lack of detection in Hodgkin’s disease. Comparison of cytogenetic analysis, reverse transcriptase-polymerase chain reaction, and P-80 immunostaining. Blood 87: 284-291. PubMed: 8547653. 8547653

[34] Pulford K, Lamant L, Morris SW, Butler LH, Wood KM et al. (1997) Detection of anaplastic lymphoma kinase (ALK) and nucleolar protein nucleophosmin (NPM)-ALK proteins in normal and neoplastic cells with the monoclonal antibody ALK1. Blood 89: 1394-1404. PubMed: 9028963. 9028963

[35] Jokoji R, Yamasaki T, Minami S, Komuta K, Sakamaki Y, Takeuchi K, Tsujimoto M (2010) Combination of morphological feature analysis and immunohistochemistry is useful for screening of EML4-ALK-positive lung adenocarcinoma. J Clin Pathol 63: 1066-1070. doi:10.1136/jcp.2010.081166. PubMed: 20935334. 20935334 10.1136/jcp.2010.081166

[36] Paik JH, Choe G, Kim H, Choe JY, Lee HJ et al. (2011) Screening of anaplastic lymphoma kinase rearrangement by immunohistochemistry in non-small cell lung cancer: correlation with fluorescence in situ hybridization. J Thorac Oncol 6: 466-472. doi:10.1097/JTO.0b013e31820b82e8. PubMed: 21258247. 21258247 10.1097/JTO.0b013e31820b82e8

[37] Yi ES, Boland JM, Maleszewski JJ, Roden AC, Oliveira AM et al. (2011) Correlation of IHC and FISH for ALK gene rearrangement in non-small cell lung carcinoma: IHC score algorithm for FISH. J Thorac Oncol 6: 459-465. doi:10.1097/JTO.0b013e318209edb9. PubMed: 21278610. 21278610 10.1097/JTO.0b013e318209edb9

[38] Park HS, Lee JK, Kim DW, Kulig K, Kim TM et al. (2012) Immunohistochemical screening for anaplastic lymphoma kinase (ALK) rearrangement in advanced non-small cell lung cancer patients. Lung Cancer 77: 288-292. doi:10.1016/j.lungcan.2012.03.004. PubMed: 22465695. 22465695 10.1016/j.lungcan.2012.03.004

[39] Rodig SJ, Mino-Kenudson M, Dacic S, Yeap BY, Shaw A et al. (2009) Unique Clinicopathologic Features Characterize *ALK*-rearranged Lung Adenocarcinoma in the Western Population. Clin Cancer Res 15: 5216-5223. doi:10.1158/1078-0432.CCR-09-0802. PubMed: 19671850. 19671850 10.1158/1078-0432.CCR-09-0802 PMC2865649

[40] Hofman P, Ilie M, Hofman V, Roux S, Valent A et al. (2012) Immunohistochemistry to identify EGFR mutations or ALK rearrangements in patients with lung adenocarcinoma. Ann Oncol 23: 1738-1743. doi:10.1093/annonc/mdr535. PubMed: 22100693. 22100693 10.1093/annonc/mdr535

[41] Wallander ML, Geiersbach KB, Tripp SR, Layfield LJ (2012) Comparison of reverse transcription-polymerase chain reaction, immunohistochemistry, and fluorescence in situ hybridization methodologies for detection of echinoderm microtubule-associated proteinlike 4-anaplastic lymphoma kinase fusion-positive non-small cell lung carcinoma: implications for optimal clinical testing. Arch Pathol Lab Med 136: 796-803. doi:10.5858/arpa.2011-0321-OA. PubMed: 22742552. 22742552 10.5858/arpa.2011-0321-OA

[42] Chen TD, Chang IC, Liu HP, Wu YC, Wang CL et al. (2012) Correlation of anaplastic lymphoma kinase overexpression and the EML4-ALK fusion gene in non-small cell lung cancer by immunohistochemical study. Chang Gung Med J 35: 309-317. PubMed: 22913857. 22913857 10.4103/2319-4170.106140

[43] Li Y, Li Y, Yang T, Wei S, Wang J et al. (2013) Clinical significance of EML4-ALK fusion gene and association with EGFR and KRAS gene mutations in 208 Chinese patients with non-small cell lung cancer. PLOS ONE 8: e52093. doi:10.1371/journal.pone.0052093. PubMed: 23341890. 23341890 10.1371/journal.pone.0052093 PMC3544857

[44] Heuckmann JM, Balke-Want H, Malchers F, Peifer M, Sos ML et al. (2012) Differential protein stability and ALK inhibitor sensitivity of EML4-ALK fusion variants. Clin Cancer Res 18: 4682-4690. doi:10.1158/1078-0432.CCR-11-3260. PubMed: 22912387. 22912387 10.1158/1078-0432.CCR-11-3260

[45] Sasaki T, Koivunen J, Ogino A, Yanagita M, Nikiforow S et al. (2011) A novel ALK secondary mutation and EGFR signaling cause resistance to ALK kinase inhibitors. Cancer Res 71: 6051-6060. doi:10.1158/0008-5472.CAN-11-1340. PubMed: 21791641. 21791641 10.1158/0008-5472.CAN-11-1340 PMC3278914

[46] Wu SG, Kuo YW, Chang YL, Shih JY, Chen YH et al. (2012) EML4-ALK translocation predicts better outcome in lung adenocarcinoma patients with wild-type EGFR. J Thorac Oncol 7: 98-104. doi:10.1097/JTO.0b013e3182370e30. PubMed: 22124476. 22124476 10.1097/JTO.0b013e3182370e30

[47] Huang SF, Liu HP, Li LH, Ku YC, Fu YN et al. (2004) High frequency of epidermal growth factor receptor mutations with complex patterns in non-small cell lung cancers related to gefitinib responsiveness in Taiwan. Clin Cancer Res 10: 8195-8203. doi:10.1158/1078-0432.CCR-04-1245. PubMed: 15623594. 15623594 10.1158/1078-0432.CCR-04-1245

[48] Wu CC, Hsu HY, Liu HP, Chang JW, Chen YT et al. (2008) Reversed mutation rates of KRAS and EGFR genes in adenocarcinoma of the lung in Taiwan and their implications. Cancer 113 supp: 3199-3208. doi:10.1002/cncr.23925. PubMed: 18932251 18932251 10.1002/cncr.23925

