# Supplementary figures and images for: Comparison of IHC, FISH and RT-PCR Methods for Detection of ALK Rearrangements in 312 Non-Small Cell Lung Cancer Patients in Taiwan
Source: PLoS One. 2013 Aug 7;8(8):e70839. doi: 10.1371/journal.pone.0070839 (PMC3737393; doi:10.1371/journal.pone.0070839)

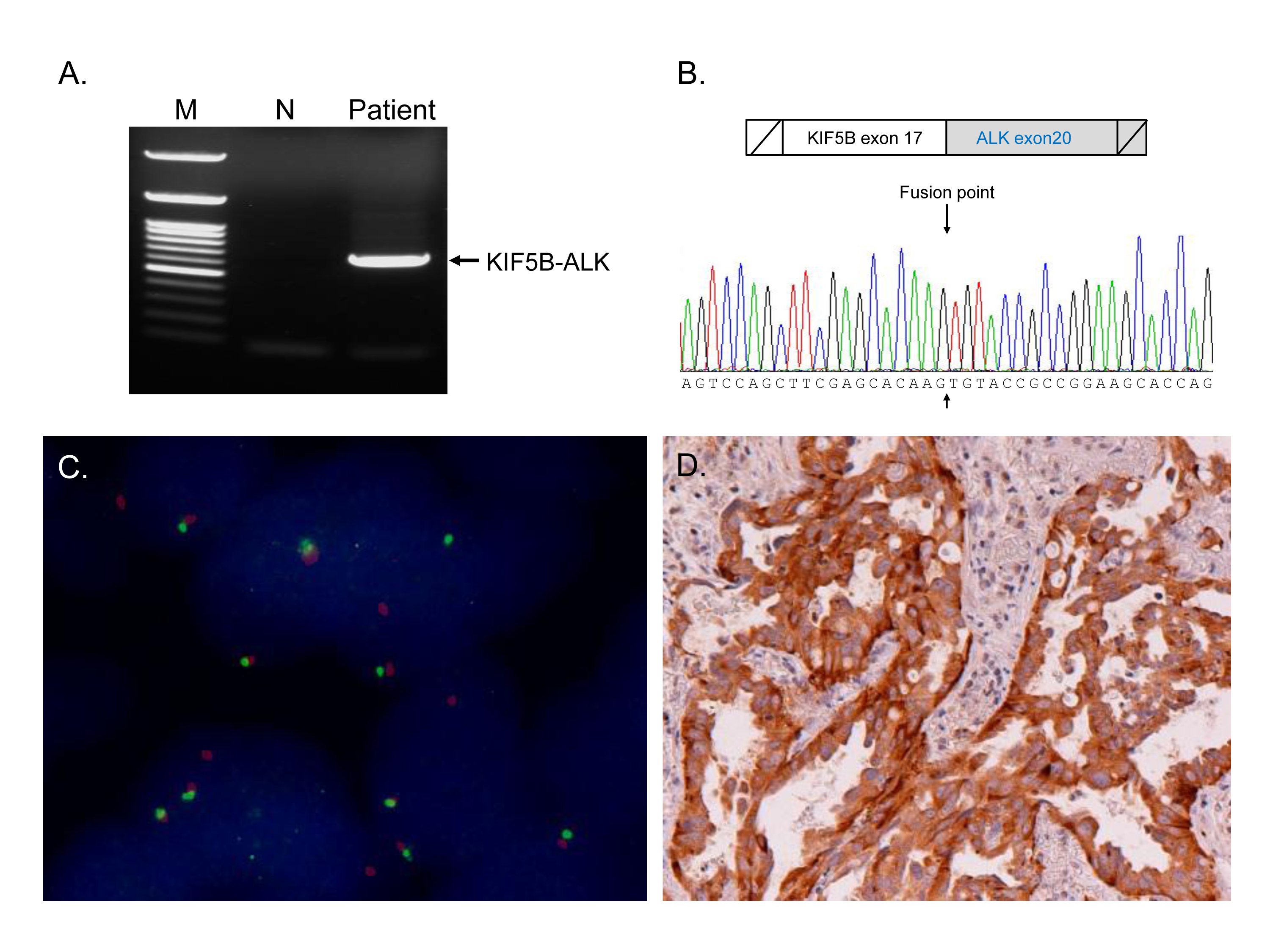

Supplement: Figure S1 — (A) Gel electrophoresis revealed correct size of the RT-PCR product. (B) The electrospherogram of KIF5B-ALK fusion gene by direct sequencing. (C) The green and red signals were wide apart for more than 2 nuclei by break-apart FISH study. (D) The IHC stain for ALK was strong (anti-ALK antibody, 200X). (TIFF) [file pone.0070839.s001.tiff]

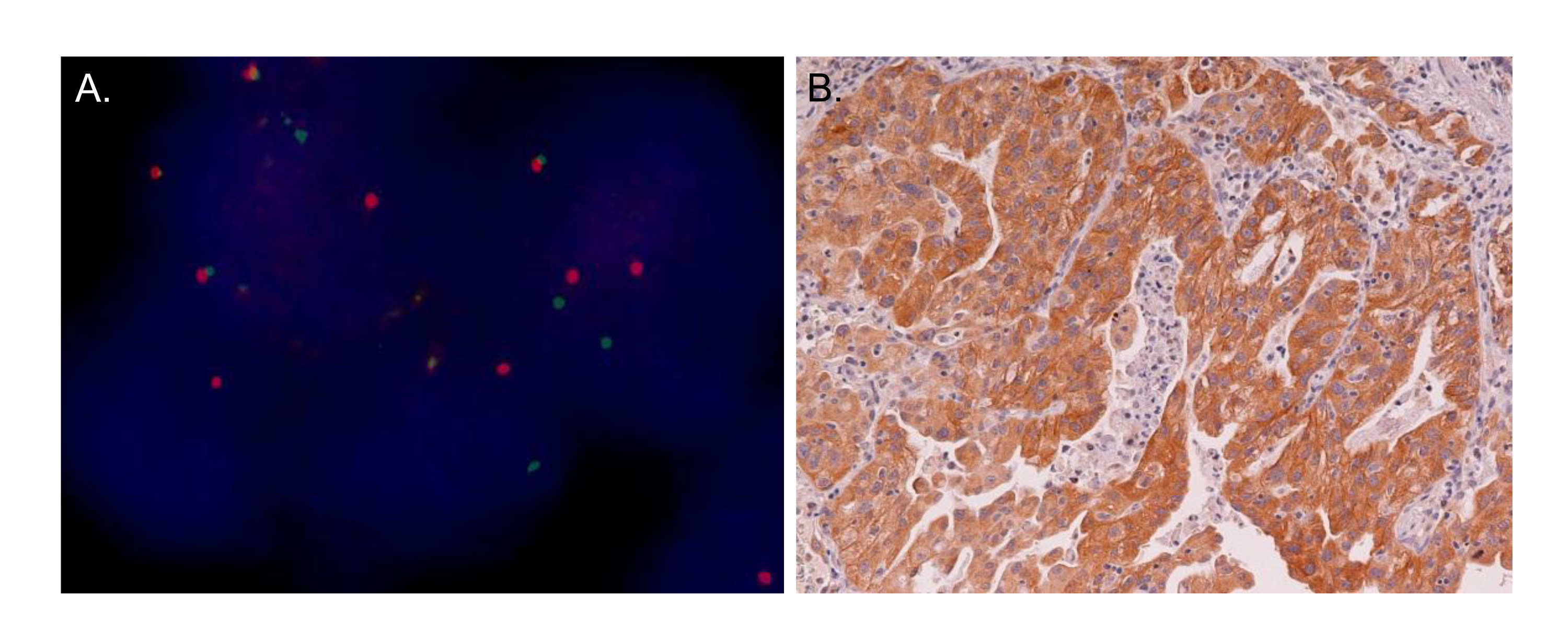

Supplement: Figure S2 — (A) The green and red signals were wide apart for more than 2 nuclei by break-apart FISH study. (B) The IHC stain for ALK was strong (anti-ALK antibody, 200X). (TIFF) [file pone.0070839.s002.tiff]

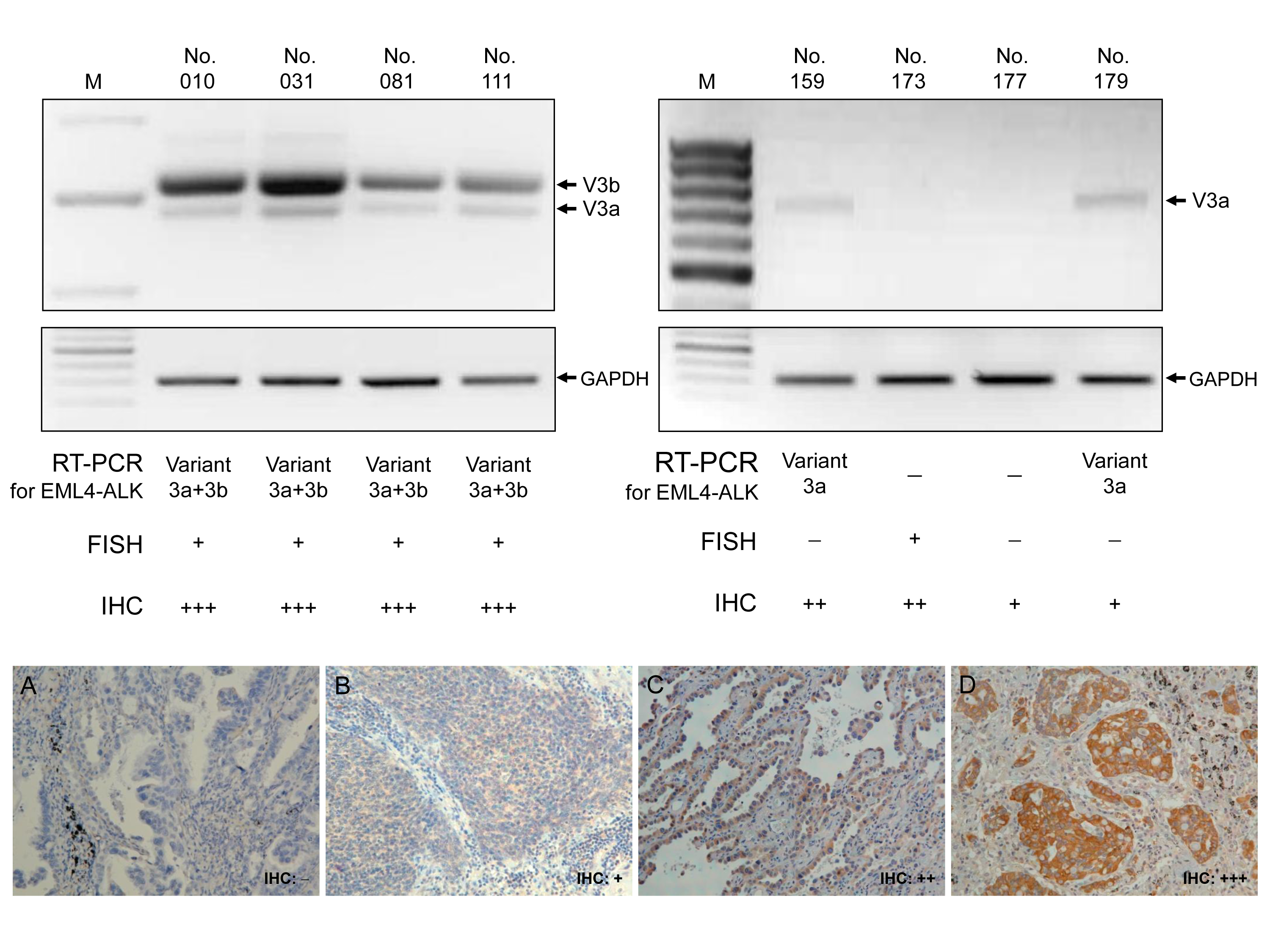

Supplement: Figure S3 — Upper left: All of the 4 patients (No. 10, 30, 81 and 111) had strong intensities of the RT-PCR products. They were all FISH (+) and IHC 3+. Upper right: Two (No. 159 and 179) of the four patients had weak intensities of the RT-PCR products, and the other two patients (No. 173 and 177) were RT-PCR (-). One of them (No. 173) was FISH (+) and IHC 2+. Lower: IHC stains for ALK in four patients are shown. (A) Patient No. 20 was totally negative for ALK, (B) Patient No. 179 (squamous cell carcinoma) was IHC 1+, (C) Patient No. 173 was IHC 2+, (D) Patient No. 111 was IHC 3+ (anti-ALK antibody, 200X). (TIF) [file pone.0070839.s003.tif]
