# Supplementary material for: Comparison of IHC, FISH and RT-PCR Methods for Detection of ALK Rearrangements in 312 Non-Small Cell Lung Cancer Patients in Taiwan
Source: PLoS One. 2013 Aug 7;8(8):e70839. doi: 10.1371/journal.pone.0070839 (PMC3737393; doi:10.1371/journal.pone.0070839)
Supplement: Table S1 — (DOCX) [file pone.0070839.s004.docx]

**Table S1. Clinical characteristics of *ALK* rearrangements detected by RT-PCR in 312 NSCLC patients**

| Variables | ALK rearrangements | | *P* values ^a^ |
| --- | --- | --- | --- |
|  | **Positive** | **Negative** |  |
| **Total number of patients** | 13 (4.17%) | 299(95.83%) |  |
| **Gender** |  |  | **0.0248** |
| **Male** | 3(1.78%) | 166(98.22%) |  |
| **Female** | 10(6.99%) | 133(93.01%) |  |
| **Median age: 64 years (range 24-87)** |  |  | 0.4040 |
| **<65 years** | 5(3.13%) | 155(96.88%) |  |
| **≥ 65 years** | 8(5.26%) | 144(94.74%) |  |
| **Smoking history** |  |  | 1.0000**^e^** |
| **Smoking-Yes (Current or Ex-smoker)** | 5(4.42%) | 108(95.58%) |  |
| **Smoking-Never** | 8(4.02%) | 191(95.98%) |  |
| **Histology type** |  |  | 0.2016 |
| **Squamous cell carcinoma** | 1(1.33%) | 74(98.67%) |  |
| **Non-squamous cell carcinoma^b^** | 12(5.06%) | 225(94.94%) |  |
| **Stage** |  |  | 0.5740 |
| **I** | 8(5.16%) | 147(94.84%) |  |
| **II** | 1(1.49%) | 66(98.51%) |  |
| **III** | 4(5.26%) | 72(94.74%) |  |
| **IV** | 0(0%) | 14(100%) |  |
| **ALK expression by IHC stain ^c^** |  |  | **<0.0001** |
| **Negative (0 or 1+)** | 1(0.42%) | 236(99.58%) |  |
| **Positive (2+ or 3+)** | 12(16.44%) | 61(83.56%) |  |
| ***ALK* fusion gene by Break apart FISH study^d^** |  |  | **<0.0001** |
| **Positive** | 7(77.78%) | 2(22.22%) |  |
| **Negative** | 5(1.69%) | 291(98.31%) |  |
| ***EGFR* mutations** |  |  | **0.0008** |
| **Mutant** | 0(0.00%) | 137(100.00%) |  |
| **Wild type** | 13(7.43%) | 162(92.57%) |  |
| ***KRAS* mutations** |  |  | 1.0000 |
| **Mutant** | 0(0.00%) | 18(100.00%) |  |
| **Wild type** | 13(4.42%) | 281(95.58%) |  |

NSCLC, non small cell lung cancer; RT-PCR, reverse transcriptase polymerase chain reaction; FISH, fluorescence in situ hybridization; IHC stain, immune-histochemical stain.

^a^By chi-square association test or Fisher’s exact test, when there is at least a cell frequency less than 5.

^b^Non-squamous cell carcinoma included 227 adenocarcinomas, 9 adenosquamous carcinomas and one carcinoid tumor.

^c^Two patients without tumor part in the tissue sections were not included.

^d^Two patients without tumor part in the tissue sections and five patients failed in FISH study were not included.

^e^By multivariate analysis, smoking history became significant (p=0.022).
