# Supplementary material for: Comparison of IHC, FISH and RT-PCR Methods for Detection of ALK Rearrangements in 312 Non-Small Cell Lung Cancer Patients in Taiwan
Source: PLoS One. 2013 Aug 7;8(8):e70839. doi: 10.1371/journal.pone.0070839 (PMC3737393; doi:10.1371/journal.pone.0070839)
Supplement: Table S2 — (DOCX) [file pone.0070839.s005.docx]

**Table S2. Comparison between RT-PCR, FISH and IHC detection methods for *ALK* rearrangement in 305 non-small cell carcinoma patients***

|  | | **Intensity of IHC stain** | | | |
| --- | --- | --- | --- | --- | --- |
| **RT-PCR** | **FISH** | **Negative** | **Weak (1+)** | **Moderate (2+)** | **High (3+)** |
| **P** | **P** | 0(0.00%) | 0(0.00%) | 0(0.00%) | 7(70.00%) |
| **P** | **N** | 0(0.00%) | 1(0.64%) | 3(4.92%) | 1(10.00%) |
| **N** | **P** | 0(0.00%) | 0(0.00%) | 1(1.64%) | 1(10.00%) |
| **N** | **N** | 76(100.00%) | 157(99.36%) | 57(93.44%) | 1(10.00%) |
| Total | | 76(100.00%) | 158(100.00%) | 61(100.00%) | 10(100.00%) |

RT-PCR, reverse transcriptase polymerase chain reaction; FISH, fluorescence in situ hybridization; IHC stain, immunohistochemical stain; P, positive; N, negative.

*****Five patients failed in FISH study (including 2 patients with negative IHC stain, one with weak intensity and two with high intensity of IHC stains) among the 310 patients with IHC data were not included in this table,.
