# Supplementary material for: Comparison of IHC, FISH and RT-PCR Methods for Detection of ALK Rearrangements in 312 Non-Small Cell Lung Cancer Patients in Taiwan
Source: PLoS One. 2013 Aug 7;8(8):e70839. doi: 10.1371/journal.pone.0070839 (PMC3737393; doi:10.1371/journal.pone.0070839)
Supplement: Table S3 — (DOC) [file pone.0070839.s006.doc]

**Table S3. Clinicopathological characteristics of 17 non-small cell lung cancer patients with ALK rearrangements or having high ALK expression**

| **No.** | **Age** | **Gender** | **Smoking**  **history** | **ALK fusion genes**  **determined By RT-PCR** | **RT-PCR**  **intensity** | **ALK**  **FISH** | **ALK**  **IHC*** | **KRAS** | **EGFR** | **Pathology diagnosis**  **(Main Histology patterns)** |
| --- | --- | --- | --- | --- | --- | --- | --- | --- | --- | --- |
| 1 | 67 | F | (+) | EML4-ALK V1 | Strong | P | 3+ | WT | WT | ADC (Papillary) |
| 2 | 25 | F | (-) | EML4-ALK V1 | Weak | N | 2+ | WT | WT | ADC (Mucinous) |
| 3 | 67 | F | (-) | EML4-ALK V1 | Weak | N | 2+ | WT | WT | ADSC |
| 4 | 67 | F | (-) | EML4-ALK V2 | Strong | P | 3+ | WT | WT | ADC (Acinar) |
| 5 | 58 | M | (+) | EML4-ALK V3a+V3b | Strong | P | 3+ | WT | WT | ADC (Papillary and acinar) |
| 6 | 50 | F | (-) | EML4-ALK V3a+V3b | Strong | P | 3+ | WT | WT | ADC (Acinar and mucinous) |
| 7 | 68 | F | (-) | EML4-ALK V3a+V3b | Strong | P | 3+ | WT | WT | ADC (Acinar and Lepidic) |
| 8 | 45 | F | (-) | EML4-ALK V3a+V3b | Strong | P | 3+ | WT | WT | ADC (Mucinous and acinar) |
| 9 | 75 | M | (+) | EML4-ALK V3a+V3b | Weak | N | 3+ | WT | WT | ADC (Acinar) |
| 10 | 75 | F | (+) | EML4-ALK V3a | Weak | N | 2+ | WT | WT | ADC (Acinar, papillary, lepidic) |
| 11 | 78 | F | (-) | EML4-ALK V3a | Weak | N | 1+ | WT | WT | SCC |
| 12 | 48 | M | (-) | EML4-ALK V3b | Strong | Fail | 3+ | WT | WT | ADC (Acinar) |
| 13 | 69 | F | (+) | KIF5B-ALK(+) | Strong | P | 3+ | WT | WT | ADC (Acinar) |
| 14 | 50 | F | (-) | Negative |  | P | 3+ | WT | WT | ADC (Papillary) |
| 15 | 58 | F | (-) | Negative |  | P | 2+ | WT | WT | ADC (Lepidic and acinar) |
| 16 | 62 | M | (-) | Negative |  | N | 3+ | WT | L858R | ADC (Acinar and papillary) |
| 17 | 73 | F | (-) | Negative |  | Fail | 3+ | WT | delE746_T751insA and V769M | ADC (Acinar, papillary, lepidic) |

RT-PCR: reverse transcription polymerase chain reaction;FISH: fluorescence in situ hybridization; IHC: immunehistochemistry; N:negative; P:positive; M:male; F:female; WT:wild type;V1: variant 1;V2:variant 2;V3a:variant 3a;V3b:variant 3b; ADC: Adenocarcinoma; ADSC:Adeno-squamous carcinoma;SCC: Squamous cell carcinoma.

*The ALK expression determined by IHC stain was grades as: 1+ (weak), 2+ (moderate), and 3+(high). .
