# Supplementary material for: Comparison of IHC, FISH and RT-PCR Methods for Detection of ALK Rearrangements in 312 Non-Small Cell Lung Cancer Patients in Taiwan
Source: PLoS One. 2013 Aug 7;8(8):e70839. doi: 10.1371/journal.pone.0070839 (PMC3737393; doi:10.1371/journal.pone.0070839)
Supplement: Table S4 — (DOC) [file pone.0070839.s007.doc]

**Table S4. Meta-analysis of 18 ALK rearrangement study reports in non-small cell lung cancer patients with RT-PCR study data**

| **No.** | **Authors [reference No.]** | **Ethnic**  **group** | **Patient sources (number)** | **Number of patients studied** | | | **Fusion genes**  **(Number)** | **Number of patients in each *EML4-ALK* Variant type** | | | | | | | |
| --- | --- | --- | --- | --- | --- | --- | --- | --- | --- | --- | --- | --- | --- | --- | --- |
|  |  |  |  | **ALK RT-PCR** | **ALK IHC** | **ALK FISH** |  | **V1** | **V2** | **V3** | **V4** | **V5** | **V6** | **V7** | **V8** |
| 1 | Soda, et al. 2007 [8] | Asian | Japan (75) | 75 | ND | ND | EML4-ALK (5) | 3 | 2 |  |  |  |  |  |  |
| 2 | Shinmura, et al. 2008 [18] | Asian | Japan (77) | 77 | ND | ND | EML4-ALK (2) | 1 | 1 |  |  |  |  |  |  |
| 3 | Inamura, et al. 2008 [19] | Asian | Japan (221) | 221 | 5 | ND | EML4-ALK (5) | 2 | 3 |  |  |  |  |  |  |
| 4 | Koivunen, et al. 2008 [21] | Caucasian Asian | U.S. (138)  Korea (167) | 305 | ND | ND | EML4-ALK (8) | 2 |  | 4(3a+3b) | 2 |  |  |  |  |
| 5  6 | Takeuchi, et al. 2008 [20]^a^  Inamura, et al. 2009 [22]^a^ | Asian | Japan (363) | 363 | 11 | 2 | EML4-ALK (11) | 3 | 3 | 3 | 1 | 1 |  |  |  |
| 7 | Takeuchi, et al. 2009 [15] | Asian | Japan (130) | 8 | 130 | 1 | EML4-ALK (7), KIF5B-ALK (1) | 1 | 1 | 3 |  |  | 1 | 1 |  |
| 8  9 | Wong, et al. 2009 [24]^b^  Wong, et al. 2011 [16]^b^ | Asian | Hong Kong (266) | 266 | 221 | 1 | EML4-ALK (13)  KIFB-ALK (1) | 2 | 2 | 8 |  | 1 |  |  |  |
| 10 | Lin, et al. 2009 [23] | Caucasian | U.S. (106) | 106 | ND | 12 | EML4-ALK (12) | 11 |  |  |  |  |  |  | 1 |
| 11 | Martelli, et al. 2009 [25] | Caucasian  Asian | Italy (481), Spain (60), Japan (101), Hong Kong (20) | 120 | 662 | 20 | EML4-ALK (9) | 7 |  | 2 |  |  |  |  |  |
| 12 | Takahashi et al. 2010 [26] | Asian | Japan (313) | 313 | ND | ND | EML4-ALK (5)^c^ | 1 |  | 1(3a),1(3b) |  |  |  |  |  |
| 13 | Zhang, et al. 2010 [27] | Asian | China (103) | 103 | ND | ND | EML4-ALK (12) | 4 | 1 | 3(3a) |  | 1 | 3 |  |  |
| 14 | Sanders, et al. 2011 [28] | Caucasian | U.S. (55) | 55 | 10 | 23 | EML4-ALK (5) | 1 |  | 3 |  |  |  |  | 1(8a+8b) |
| 15 | Hofman et al. 2012 [40] | Caucasian | France (154) | 19 | 20 | 20 | EML4-ALK (3)^d^ |  |  |  |  |  |  |  |  |
| 16 | Wallander, et al.2012 [41] | Caucasian | U.S. (46) | 46 | 46 | 46 | EML4-ALK (11)^e^ | 9 |  | 2(3a+3b) |  |  |  |  |  |
| 17 | Li, et al. 2013 [43] | Asian | China (208) | 208 | 7^c^ | ND | EML4-ALK (7) | 2 | 1 | 4 |  |  |  |  |  |
| 18 | Present report | Asian | Taiwan (312) | 312 | 310 | 305 | EML4-ALK (12)  KIFB-ALK (1) | 3 | 1 | 2(3a), 1(3b), 5(3a+3b) |  |  |  |  |  |

RT-PCR: reverse transcriptase-polymerase chain reaction; FISH: fluorescence in situ hybridization; IHC: Immunohistochemical stain; ND: not done..

^a^ There were overlapping in the patient sources for No.5 & 6 reports, so the data shown were the pooling together results.

^b^ There were overlapping in the patient sources for No.8 & 9 reports, so the data shown were the pooling together results.

^c^ Two of the 5 patients positive for EML4-ALK by RT-PCR study had no variant type data (the authors considered they might be novel types).

^d^ This study did not give the data of EML4-ALK variant types.

^e^ All 46 patients were adenocarcinoma, and 42 patients (42/46,91%) were known to be wild type for epidermal growth factor receptor gene.
