# Supplementary material for: Comparison of IHC, FISH and RT-PCR Methods for Detection of ALK Rearrangements in 312 Non-Small Cell Lung Cancer Patients in Taiwan
Source: PLoS One. 2013 Aug 7;8(8):e70839. doi: 10.1371/journal.pone.0070839 (PMC3737393; doi:10.1371/journal.pone.0070839)
Supplement: Table S5 — (DOC) [file pone.0070839.s008.doc]

**Table S5. The ethnicities and variant types of EML4-ALK fusion gene among 2273 NSCLC patients***

| **Ethnic**  **group** | **Patient sources (number)** | **ALK Fusion gene** | | **EML4-ALK Variant type (Number of patients)** | | | | | | | |
| --- | --- | --- | --- | --- | --- | --- | --- | --- | --- | --- | --- |
|  |  | **KIF5B-ALK** | **EML4-ALK** | **V1** | **V2** | **V3** | **V4** | **V5** | **V6** | **V7** | **V8** |
| **Asian** | Japan (1057) | 1 | 33 | 11 | 10 | 8 | 1 | 1 | 1 | 1 | 0 |
|  | China (311)** | 0 | 19 | 6 | 2 | 7 | 0 | 1 | 3 | 0 | 0 |
|  | Hong Kong (266)** | 1 | 13 | 2 | 2 | 8 | 0 | 1 | 0 | 0 | 0 |
|  | Taiwan (312)** | 1 | 12 | 3 | 1 | 8 | 0 | 0 | 0 | 0 | 0 |
| **Total for Asian** | **1946** | **3** | **77** | **22** | **15** | **31** | **1** | **3** | **4** | **1** | **0** |
| **Caucasian** | Italy and Spain (120) | 0 | 9 | 7 | 0 | 2 | 0 | 0 | 0 | 0 | 0 |
|  | U.S.(207) | 0 | 28 | 21 | 0 | 5 | 0 | 0 | 0 | 0 | 2 |
| **Total for Caucasian** | **327** | **0** | **37** | **28** | **0** | **7** | **0** | **0** | **0** | **0** | **2** |

*This was the summary data of the 16 study reports listed in Table 4. Two studies (No. 4 and No.15) were not included, since the ethnicity or variant types were not found in their reports.

**Variant 3 was the most common type in Chinese population (23/44, 52.3%), while Variant 1 was most common in Caucasian (28/37, 75.7%). The difference was statistically significant for both Variant 1(p= 0.0000) and for Variant 3 (p=0.0020)
