# Supplementary material for: Comparison of IHC, FISH and RT-PCR Methods for Detection of ALK Rearrangements in 312 Non-Small Cell Lung Cancer Patients in Taiwan
Source: PLoS One. 2013 Aug 7;8(8):e70839. doi: 10.1371/journal.pone.0070839 (PMC3737393; doi:10.1371/journal.pone.0070839)
Supplement: Table S6 — (DOCX) [file pone.0070839.s009.docx]

**Table S6. Primer sets for detection of ALK rearrangements**

| **ALK**  **rearrangements** | **Primers** | |
| --- | --- | --- |
|  | **Forward** | **Reverse** |
| ***EML4-ALK*** |  |  |
| **Variant 1** | **5'-GTGCAGTGTTTAGCATTCTTGGGG-3'** | **5'-TCTTGCCAGCAAAGCAGTAGTTGG-3'** |
| **Variant Non-1** | **5'-GTCAGCTCTTGAGTCACGAGTT-3'** | **5'-TCTTGCCAGCAAAGCAGTAGTTGG-3'** |
|  |  |  |
| ***KIF5B-ALK*** | **5'-TCGGCAACTTTAGCGAGTA-3'** | **5'-GGACACCTGGCCTTCATAC-3'** |
|  |  |  |
| ***KLC1-ALK*** | **5'-ATGTATGACAACATGTCCAC-3'** | **5'-TCAGGGCCCAGGCTGGTTCA-3'** |
|  |  |  |
| ***TFG-ALK*** | **5'-TCGTTTATTGGATAGCTTGGAACCAC-3'** | **5'-TCTTGCCAGCAAAGCAGTAGTTGG-3'** |
